# Household income determines access to specialized pediatric chronic pain treatment in Germany

**DOI:** 10.1186/s12913-016-1403-9

**Published:** 2016-04-21

**Authors:** Ann-Kristin Ruhe, Julia Wager, Gerrit Hirschfeld, Boris Zernikow

**Affiliations:** German Paediatric Pain Centre, Children’s and Adolescents’ Hospital, Datteln, and Department of Children’s Pain Therapy and Paediatric Palliative Care, Witten/Herdecke University, Faculty of Health – School of Medicine, Dr.-Friedrich-Steiner-Str .5, 45711 Datteln, Germany; Faculty of Economics and Social Sciences, University of Applied Sciences Osnabrück , Caprivistr. 30A, 49076 Osnabrück, Germany

**Keywords:** Socioeconomic status (SES), Pediatric, Chronic pain, Health care delivery, Income, Inequality, Specialized treatment

## Abstract

**Background:**

Families with lower socioeconomic status (SES) often face problems with gaining access to health care services. Information is scarce on the relationship between SES and health care delivery for children suffering from chronic pain.

**Methods:**

Families presenting to a specialized pain center (*N* = 1,001) provided information on ‘household income, ‘parental education’ and ‘occupation’ to aid the evaluation of their SES. To assess whether the SES of the clinical sample is representative of the general population, it was compared to data from a community sample (*N* = 14,558). For the clinical sample, travel distance to the clinic was described in relation to the 75 % catchment area. Multiple logistic regression was used to analyze the association between SES and the journey from outside the catchment area.

**Results:**

The SES was significantly higher in the clinical sample than in the community sample. Within the clinical sample, the distance traveled to the pain center increased with increasing SES. The 75 % catchment area was 143 miles for families with the highest SES and 78 miles for the lowest SES. ‘Household income’ predicted travel distance (OR 1.32 (1.12–1.56)). Education and occupational status were not significant predictors of travel from outside the catchment area.

**Conclusions:**

In Germany, specialized care for children with chronic pain is subject to disparities in access. Future activities should focus on identifying barriers to access and seeking to prevent inequalities in specialized pediatric health care delivery. Increasing the number of specialized treatment facilities could improve access to specialized pediatric pain treatment, regardless of socioeconomic determinants.

**Electronic supplementary material:**

The online version of this article (doi:10.1186/s12913-016-1403-9) contains supplementary material, which is available to authorized users.

## Background

One important goal of developed societies is that every citizen has access to good health care [1–3]. However, barriers to health care access exist. They range from regionally reduced availability of services (i.e., travel distances [4, 5]) and unaffordable treatment costs to a lack of information about treatment options [6].

Irrespective of the health care system, children already experience inequalities in health care use due to social differences and poverty. For example, German families with a lower socioeconomic status (SES) consult health care specialists, like pediatricians, ophthalmologists and dermatologists, less often than families with a higher SES [7]. In the US, children from families with lower household income in particular seem to have difficulties in accessing specialized care [8, 9]. Children with chronic health complaints also experience these social inequalities in different health care systems [6, 10, 11].

Comparability across studies dealing with the role of SES in health care utilization is generally hampered due to differences in sample characteristics, health care systems (i.e., type of regulation, service provision and financing [12]) or SES measures [13]. Studies often do not apply a comprehensive measure of SES that includes household income, parental education and occupation [5, 14–19].

In children, chronic pain is a major health care problem [20] that requires treatment to interrupt chronification [21]. However, it often takes a long time for these children to reach a specialized care center [22]. To our knowledge, only one study thus far has investigated the possible effects of SES on health service use in children with chronic pain [5]. This German study showed that children of parents with a higher occupational skill level traveled longer distances to receive specialized pain treatment [5]. Other socioeconomic factors were not investigated in this study. In adult chronic pain patients, few studies have analyzed the determinants of health care use. These showed that access to health care is associated with higher socioeconomic factors such as income or education [23, 24].

The aim of this study is to explore the effect of SES on health care utilization at a specialized pediatric pain center in Germany. SES was measured as a multidimensional construct including ‘household income,’ ‘parental education’ and ‘parental occupation’. This measure has previously been applied by a German community survey [25, 26]. The current study specifically investigated the following aspects: 1) comparability of the SES between a clinical pain sample and a community sample; 2) the association between SES and distance traveled to a specialized pediatric pain center; 3) the impact of single socioeconomic parameters on the distance traveled to a specialized pediatric pain center in Germany.

## Methods

### Data sources

We analyzed data from two samples of German children and adolescents aged between 3 and 17 years: a community sample (*N* = 14,558) and a clinical sample (*N* = 1,001) from a specialized pediatric pain clinic.

The community sample comprises a representative study sample from the German Health Interview and Examination Survey for Children and Adolescents (KiGGS), conducted by the German Robert Koch Institute (RKI) from May 2003 to May 2006. A Public Use File of the KiGGS-data is available at the RKI. Data on SES were missing for 278 subjects (drop-out rate: 1.9 %). In the community sample, half of the children (51 %) were male, and the mean age was 10 years (SD = 4.2). A detailed description of the sample and the study design has been provided elsewhere [27–29].

The clinical sample comprises children and adolescents who sought treatment at the German Paediatric Pain Centre (GPPC) between August 1, 2012, and March 31, 2014 (a period of 18 months). Of the original 1037 patients, 30 families did not fill out the SES questionnaire and 6 children were excluded because they reported living in an institution or with relatives, making assessment of parental SES unfeasible (drop-out rate: 3.5 %). The majority of the 1001 children in the clinical sample were female (67 %), and the mean age was 13 years (SD 2.9). The most frequent main pain locations were the head (58 %), back/extremities (26 %) and abdomen (24 %).

### Measures

In the community sample, adolescents aged between 11 and 17 years completed their own questionnaire of pain items. For those under the age of 11, parents answered the questions [28]. We extracted basic demographic information (e.g., age, sex) and information on the pain problem: *any pain* during the previous three months, *recurrent pain* (i.e., pain that occurs at least once in a month for more than the previous three months) and *≥1 physician contact,* i.e., children and adolescents who reported at least one physician contact due to their recurrent pain problem. SES was measured on the basis of parental information regarding their education and vocational training, their occupational situation and the household income per month, by means of a standardized questionnaire. The respective level of these three single parameters was categorized by the assignment of value points ranging from 1 (lowest level) to 7 (highest level). Using this information, the SES could be operationalized into a multidimensional status index called the Winkler Index, which is a continuous measure (scale range 3–21) [26]. A detailed description of level operationalization has been published [30]. If information on education and occupation was available for both parents, the highest level provided was used to define parental education and occupation. Additionally, the socioeconomic status level was divided into three status groups: low (range 3–8), medium (range 9–14) and high (15–21) [30].

In the clinical sample, a battery of questionnaires was completed prior to the first appointment. In adolescents aged 11 years and older, information on the pain issue was measured by self-report, while the information was gained by parent proxy report for children younger than 11 years of age. For the clinical sample, the following data were collected: basic demographic information (e.g., age, sex, place of residence), information on the pain issue (e.g., pain intensity and frequency, pain-related school absence and disability and main pain location head, abdomen, back/extremities]), as well as the number of physicians/therapists’ consultations during the past three months. The impairment level (extremely high vs. high) was determined by means of predefined indicators for intensive inpatient pain treatment [22]. Children had to fulfill three of the following five criteria to be considered extremely highly impaired:

- pain duration ≥6 months,
- constant pain with an average pain intensity of NRS ≥5,
- pain peaks of NRS ≥8,
- at least 5 days of absence from school within the preceding 4 weeks
- high pain disability (P-PDI ≥36) [31–33].

In the clinical sample, parents also reported on their own education and vocational training, their occupational situation and the household income per month, and these factors were used to calculate the Winkler Index [26].

In the present study, access refers to the actual usage of a specialized pediatric pain clinic. It was measured by means of travel distance (in miles and kilometers). Travel distance to the clinic was determined based on the patients’ zip codes and calculated using Google Maps. Travel distance was described in relation to the 75 % catchment area, i.e., the area that included 75 % of the patients [34]. In addition, parents reported on their means of transportation (i.e., car, motorcycle, bicycle, public transport, taxi, or on foot).

### Ethics

The Ethics committee of the Children’s Hospital in Datteln, Germany, approved the study. At the time of the initial presentation, all families gave written informed consent for data collection, electronic storage and data analysis.

### Statistical methods

Patient characteristics, socioeconomic status and travel distance were presented using descriptive statistics including frequencies, means, and standard deviations [SDs]. The socioeconomic differences between the community and the clinical sample were tested with Student’s t-test (for the Winkler Index) and the Mann-Whitney-U test (for the three SES status groups household income, parental education and occupation). Effect sizes were calculated and interpreted according to established standards [35].

To test the association between SES and travel distance in the clinical sample, we performed two different analyses. First, we calculated the differences in the 75 % catchment area between SES status groups. The 95 % confidence intervals (CI) were generated using bootstrapping (with 1000 replications). Second, a multiple logistic regression model calculated the Odds Ratio (OR) (of the Winkler Index) for coming from outside the 75 % catchment area. Control variables were included based on previous research [5] as follows: the child’s age, sex, impairment level (extremely high vs. high impairment), the main pain location (head, abdomen or back/extremities), and the number of physicians/therapists’ consultations during the past three months (as a continuous variable). To allow comparisons across regression coefficients, the three continuous variables ‘age’, ‘number of consultations’ and the ‘Winkler Index’ were standardized, i.e., divided by twice the SD [36]. The relative contribution of each factor associated with high travel distance was quantified as an OR with a 95 % CI. The significance level was set at *p* < 0.05.

To work out the sole impact of the three single socioeconomic parameters of the Winkler Index, a multivariate logistic regression model was constructed, including *income* (seven income groups), *educational level* (seven categories with value points from 1 to 7) and *occupational status* (seven categories with value points from 1 to 7). The influencing factors mentioned above were included in the logistic regression model.

All analyses were performed using SPSS Version 22.0.

## Results

### Socioeconomic status - community versus clinical sample

In the community sample, one-quarter of the families (26 %) reported a high SES compared with nearly one-half (45 %) in the clinical sample. More families in the community sample had low SES (28 %) compared with the clinical sample (10 %). Accordingly, the mean SES in the clinical sample was significantly higher (Winkler Index: 13.9 versus 11.5). The strength of the socioeconomic difference between the clinical sample and the community sample was high (Cohen’s d: 0.6) [35] (Table 1).

**Table 1:** Socioeconomic status in the community and the clinical sample

	Clinical Sample	Community Sample			
	All	All	Children with any pain^a^	Children with recurrent pain^b^	Children with ≥ 1 physician contact due to recurrent pain^c^
	*n* = 979	*n* = 14,455	*n* = 9901	*n* = 3340	*n* = 2244
Winkler-Index (range 3 – 21)					
Mean	13.9	11.5	11.5	11.5	11.1
SD	4.1	4.3	4.3	4.3	4.2
Mean difference		-2.5***	−2.4***	−2.4***	−2.8***
Effect Size (Cohen’s d)		0.6	0.6	0.6	0.7
SES status group					
Low (3 – 8)	9.7 %	27.5 %	26.9 %	26.3 %	29.5 %
Medium (9 – 14)	45.5 %	46.9 %	47.4 %	48.3 %	48.4 %
High (15 – 21)	44.8 %	25.6 %	25.7 %	25.4 %	22.2 %
* p* ^*#*^		<0.001	<0.001	<0.001	<0.001

comparisons were calculated for all subgroups of the community sample with the clinical sample

***p<0.001 (Student’s t-test). ^#^ *p*-value for Mann-Whitney-U test

^a^Any pain: children and adolescents reporting any pain during the last three months

^b^Recurrent pain: children and adolescents reporting recurrent pain, i.e., pain that occurs at least once in a month for more than the previous three months

^c^≥1 Physician contact due to recurrent pain: children and adolescents who reported at least one physician contact due to their recurrent pain problem

The analyses comparing the pain subgroups in the community sample with the clinical sample regarding socioeconomic differences revealed similar results: Irrespective of whether children in the community sample reported any pain or recurrent pain and whether they had previous physician contact due to pain, the mean SES in the clinical sample was significantly higher (Table 1).

**Fig. 1:**
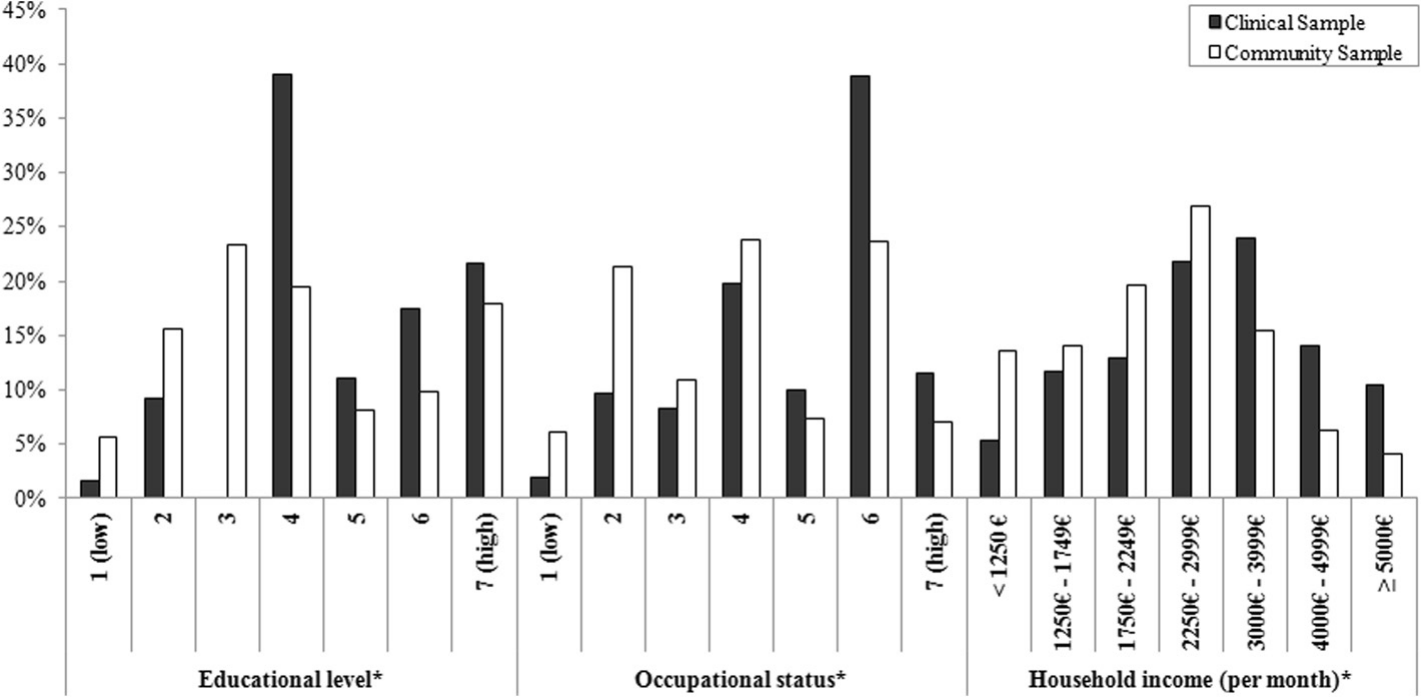
Distribution of socioeconomic parameters. *Differences between the clinical and the community sample were statistically significant with *p*<0.001 (Mann-Whitney-U test)

The distribution of the three single socioeconomic parameters revealed that parents within the clinical sample had higher educational and occupational levels as well as household income compared with the parents in the community sample (*p* < 0.001) (Fig. 1).

### Socioeconomic status and its association with travel distance (in the clinical sample)

Families in the clinical sample traveled an average of 86 miles (range: 1.5 – 434 miles), or 138 km (range: 2.3 – 698 km), to the GPPC. The 75 % catchment area spanned 121 miles (195 km). Most families (95 %) traveled by car.

A significant association was found between SES and distance traveled (*p* < 0.001), with the higher SES status group (based on the Winkler Index) traveling a longer distance to the clinic.

**Fig. 2:**
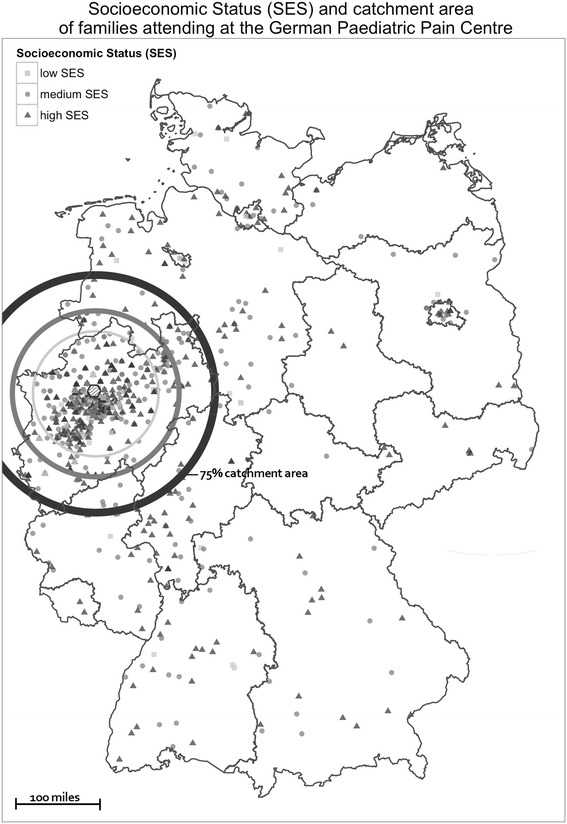
Socioeconomic status (SES) and catchment area of families attending at the German Paediatric Pain Centre

The 75 % catchment areas for families with low and medium socioeconomic status were 78 miles (95 % CI: 46.2–130.0), or 125 km, and 100 miles (95 % CI: 90.9–123.0), or 161 km, respectively. Families with a high socioeconomic status had a 75 % catchment area of 143 miles (95 % CI: 125.8–162.6), or 231 km (Fig. 2).

The logistic regression model revealed that families were more likely to come from outside the 75 % catchment area (>121 miles) as their socioeconomic status increased (OR 1.79 (1.2–2.68)). In considering the three single parameters of SES within multiple logistic regression, the families’ household income was the only factor that was significantly associated with travel distance, i.e., with increasing household income, the probability of coming from outside the catchment area increased (OR 1.32 (1.12–1.56)). Parental education and occupational status had no significant effect (OR 0.94 (0.79–1.12) and OR 1.01 (0.86–1.21), respectively).

Accordingly, the 75 % catchment area for families with a household income up to 2249 Euro per month was only 92 miles (95 % CI: 71.1–125.9), or 148 km. Families with a household income between 2250 Euro and 3999 Euro per month had a 75 % catchment area of 113 miles (95 % CI 95.3–130.0), or 182 km, and those with a household income over 4000 Euro per month had a 75 % catchment area of 153 miles (95 % CI 135.6–189.9), or 246 km.

## Discussion

Children seeking a specialized pediatric pain clinic have a higher SES compared with a community sample. Within the clinical sample, children of parents with a higher SES traveled longer distances to the pain clinic than did children of parents with a lower SES. The 75 % catchment area for families with a low SES amounted to almost half (78 miles) the distance in the high SES group (143 miles). Of the three single socioeconomic parameters, income has the single most significant association with travel distance.

In line with the results of the current study, previous studies in children and adults have revealed that low SES is a barrier for health care utilization (see Additional file 1: Table S1 and Additional file 2: Table S2). Different reasons for this situation have been proposed. In some countries, not all children and their families have health insurance, and there can also be financial gaps in private or public programs that may influence the use of health care services or access, especially among low-income families [37]. Furthermore, a lack of information can be a barrier to accessing health care [6], especially in countries without institutionalized gate keeping systems, such as Germany, where patients can decide themselves which physician they wish to consult. Finally, barriers for patients with chronic pain may include limitations in the availability of services [38, 39].

The results of the present study showed that the combined measure of the Winkler-Index has a stronger association with travel distance than the three single parameters of the SES. Accordingly, the combined measure seems to be the most validated and reliable representation of the overall construct of SES.

The present study also depicted the individual effects of income, parental education and occupation. Surprisingly, only income, not educational or occupational status, showed a significant association with the distance traveled. In Germany, people are generally obliged to obtain state or private health insurance coverage. Only 0.2 % of the German working population lacks health insurance. Usually children are covered under their parents’ insurance [40] and the insurant is eligible for free health care services for disease prevention and treatment [41], including specialized pain therapy. These results suggest that children of parents with a lower income face significant barriers to accessing appropriate care. Because health insurance covers treatment costs, the financial situation of the family seems to determine whether they can afford the journey to the pain center, which is not covered by their health insurance.

Importantly, interpretations of the results need to take into account the socioeconomic imbalance between the community and the clinical sample. SES in the clinical sample is not representative of the entire German population. Those families presenting at the specialized pain clinic report higher educational and occupational levels, as well as disposable income. Those families with a low social status in particular are underrepresented. Therefore, any striking differences within the clinical group might not be obvious, as the SES level is quite high within this group. In consideration of this selection bias, it is not clear whether education and occupational status may play a role among the population in general and whether the income gradient is even greater.

Due to the higher SES in the clinical sample, it could be assumed that severe chronic pain is more prevalent with increasing SES. Current studies, however, provide no evidence that the socioeconomic level is a predictor for the development of chronic pain [42–44]. Thus, there seems to be no increased demand (i.e., greater disease severity or prevalence) in some status groups that could explain the greater effort higher status groups make in terms of traveling longer distances. For families with a lower SES in particular, the insufficient supply of specialized pediatric pain services seems to have a negative impact on access.

### Strengths of this study

Information on SES has been collected from a large number of families visiting a specialized pain clinic. To our knowledge, there is no comparable study in the literature that analyzes the effect of SES on health care utilization in such a large pediatric chronic pain sample. The drop-out rate within this study is very low, which allows for good generalizability for similar institutions. Furthermore, this low drop-out rate shows that parents are willing to provide sensitive information for the sake of a research project that may lead to benefits for patients.

This study operationalized SES for the purposes of an epidemiological study. A frequent problem with tertiary care research is the lack of comparability with the general population. This approach allowed us to compare clinical data with general population data.

### Limitations

There are some limitations that must be kept in mind when interpreting the present findings.

First, it is important to note that health care utilization is influenced by supply and (individual or community) demand [45]. Hence, the association between SES and health service utilization is more complex than the present study implies. There are further potential factors beyond SES and those influencing variables that were controlled for that may influence a patient’s decision to seek treatment, including the quality and type of care, as well as time resources [46, 47] and the willingness to seek treatment [45]. It could be that further influencing factors such as lifestyle and living conditions need to be taken into account to evaluate the unequal distribution of health risks and health-related opportunities [48]. Second, in this study, we used travel distance calculated by zip codes as a measure of access. This, however, is only one possible criterion to describe access. An alternative measure could be travel burden, costs or actual travel time. The use of zip codes to calculate travel distance furthermore failed to take into account the actual travel distance. It only represents a rough estimate. A more precise determination would require the family’s full address.

Third, data from the clinical sample only offer information on those children and adolescents seeking treatment at the German Paediatric Pain Centre. This clinic is unique in Germany, offering inpatient treatment on a ward solely for chronic pain. However, this study does not capture data from children and adolescents suffering from chronic pain who have a need for specialized treatment but who fail to reach appropriate care due to socioeconomic barriers, undersupply or other influencing factors. The closest we could get to this group was by comparing the clinical sample with those children from the general population seeking care due to recurrent pain.

Finally, results of the present study cannot be generalized to all countries, because they highly depend on the health care system and the availability of services.

## Conclusion

The results of the present study suggest that German children and adolescents suffering from chronic pain are subject to disparities in access to specialized health care. Within the clinical sample, children from high-income families were much more likely to come from afar. The large catchment area may indicate inadequate resources in other regions. Therefore, if the long travel distances and subsequent costs are the main problem, providing a greater number of treatment facilities could be one way to enhance health care delivery for children and adolescents with chronic pain. Future research needs to examine the gaps in the knowledge base with regards to barriers to care, especially the broader patterns of socioeconomic factors and their association with resource utilization in the general population. A better understanding of the patterns of socioeconomic gradients in children’s health could identify vulnerable groups and inform policy in terms of barriers to equal delivery and access. This knowledge may form the basis for the development of measures that may be protective and supportive for socially disadvantaged children and their families.

## Additional files

Additional file 1: Table S1. Socioeconomic status and access to health care services (studies in children and adolescents) [5, 6, 8–11, 49–51]. (DOC 52 kb) Additional file 2: Table S2. Socioeconomic status and access to health care services in adult chronic pain patients [23, 24, 52]. (DOC 37 kb)

## Abbreviations

CI: confidence interval
SES: socioeconomic status
GPPC: German Paediatric Pain Centre
KiGGS: German Health Interview and Examination Survey for Children and Adolescent
km: kilometer
NRS: numeric rating scale
OR: odds ratio
P-PDI: pediatric pain disability index
RKI: Robert Koch Institute
SD: standard deviation
