# Supplementary material for: Household income determines access to specialized pediatric chronic pain treatment in Germany
Source: BMC Health Serv Res. 2016 Apr 21;16:140. doi: 10.1186/s12913-016-1403-9 (PMC4840873; doi:10.1186/s12913-016-1403-9)
Supplement: Additional file 1: Table S1. — Socioeconomic status and access to health care services (studies in children and adolescents) [5, 6, 8–11, 49–51]. (DOC 52 kb) [file 12913_2016_1403_MOESM1_ESM.doc]

**Table S1 Socioeconomic status and access to health care services (studies in children and adolescents)**

| **Author (year)** | **Sample** | **Methods** | **Country** | **Main results** |
| --- | --- | --- | --- | --- |
| (Allin and Stabile, 2012) | Community sample  Age: 1–15 years;  N= 13439 households (N=66291 observations due to follow-up) | Longitudinal survey (1994/95–2008/09) with assessments every two years;  questionnaires (children and parents) | Canada | Lower income families use more curative care compared with families with higher income, who use both preventive and curative care. |
| (Amone-P'Olak et al., 2010) | Community sample,  Age: 12-15 years;  N=2149 | Cross-sectional study, questionnaire (parents) | Dutch | Higher educational level of the mother as well as higher socioeconomic position are associated with better accessibility to specialized mental health services, irrespective of the severity of mental health complaints. |
| (Carlisle et al., 2012) | Clinical sample (psychiatric disorders)  Age: 15- 19 years  N= 7111 | Cross-sectional study, health administrative databases | US | Adolescents with high socioeconomic status were more likely to use aftercare services. |
| (Kemper et al., 2013) | Community sample (with mental health conditions)  Age: 7-17 years  N= 5651 | Cross-sectional study,  face-to-face with parents | US | Higher parental education and higher household income were associated with increased use of complementary and alternative medical therapies. |
| Kuhlthau et al., 2004) | Community sample  Age: 2-17 years  N=11338 | Cross-sectional study,  questionnaire (parents) | US | Lower financial resources and lower parental educational status were associated with less utilization of specialist care. |
| (Larson and Halfon, 2010) | Community sample  Age: 0-17 years  N= 102353 | Cross-sectional study,  telephone interview (parents) | US | Low income reduced access to health care services. |
| (Valenzuela et al., 2014) | Clinical sample (Type 1 diabetes)  Age: <20 years  N=780 | Cross-sectional study,  questionnaire (parents) | US | Low income was associated with reduced access to specialized diabetes care due to barriers such as cost, communication and information. |
| (Wager et al., 2013) | Clinical sample (chronic pain)  Age: 0-20 years  N=2248 | Cross-sectional study,  questionnaires (children and parents) | Germany | Children of parents with a high occupational skill level traveled longer distances to receive specialized pain treatment. |
| (Wisk and Witt, 2012) | Community sample  Age: 0-17 years  N=14138 | Cross-sectional study,  interview (parents) | US | Lower income was associated with delayed or forgone care. |
