# Supplementary material for: Household income determines access to specialized pediatric chronic pain treatment in Germany
Source: BMC Health Serv Res. 2016 Apr 21;16:140. doi: 10.1186/s12913-016-1403-9 (PMC4840873; doi:10.1186/s12913-016-1403-9)
Supplement: Additional file 2: Table S2. — Socioeconomic status and access to health care services in adult chronic pain patients [23, 24, 52]. (DOC 37 kb) [file 12913_2016_1403_MOESM2_ESM.doc]

**Table S2 Socioeconomic status and access to health care services in adult chronic pain patients**

| **Author (year)** | **Sample** | **Methods** | **Country** | **Main results** |
| --- | --- | --- | --- | --- |
| (Azevedo et al., 2013) | Community sample  Age: >18 years;  N=5094 | Cross-sectional study, telephone interviews | Portugal | Low educational level was associated with lower utilization of non-pharmacological treatment methods. Overall, socioeconomic factors were important determinants for health service use. |
| (Nguyen et al., 2005) | Community sample  Age: >18 years;  N=454 whites, 447 African-American, 434  Hispanics | Cross-sectional study,  telephone interview | US | Access to health care services was lower in low-income adults or people with lower educational status. |
| (Rahman et al., 2011) | Clinical sample (patients with osteoarthritis)  Age: > 20 years  N=34420 | Longitudinal study (1991-2004),  population-based administrative data | Canada | Patients with higher socioeconomic status were more likely to undergo artificial joint replacements and surgical consultations. |
